# The need and benefit of immune monitoring to define patient and disease heterogeneity, mechanisms of therapeutic action and efficacy of intervention therapy for precision medicine in type 1 diabetes

**DOI:** 10.3389/fimmu.2023.1112858

**Published:** 2023-01-17

**Authors:** Bart O. Roep

**Affiliations:** Department of Internal Medicine, Section of Immunomodulation and Regenerative Cell Therapy, Leiden University Medical Center, Leiden, Netherlands

**Keywords:** immunotherapy, type 1 diabetes, immune intervention, precision medicine, immune monitoring, autoimmune disease, disease endotypes, mechanism of action

## Abstract

The current standard of care for type 1 diabetes patients is limited to treatment of the symptoms of the disease, insulin insufficiency and its complications, not its cause. Given the autoimmune nature of type 1 diabetes, immunology is critical to understand the mechanism of disease progression, patient and disease heterogeneity and therapeutic action. Immune monitoring offers the key to all this essential knowledge and is therefore indispensable, despite the challenges and costs associated. In this perspective, I attempt to make this case by providing evidence from the past to create a perspective for future trials and patient selection.

## Introduction

The type 1 diabetes (T1D) community has been blessed with an impressive gain in insight into the immunopathogenesis of T1D in recent years. Detection of islet autoantibodies has been standard of practice to confirm a diagnosis of T1D or to predict future development of this disease (1). Islet autoantibodies have proven unambiguous, robust and specific immune correlates of risk and development of T1D (2). With the autoantibody targets (pro)insulin, zinc-transporter 8 (ZnT8), glutamic acid decarboxylase 65kDa (GAD65) and insulinoma antigen-2 (IA-2) being intracellular and thus being inaccessible, the conundrum of autoantibodies in relation with T1D has not yet been resolved and a pathogenic role remains elusive. Accordingly, their role as correlates of successful immune intervention has been disappointing: preserved beta-cell function so far does not associate with changes in islet autoantibody appearance in immune intervention trials of T1D, despite many efforts (3). This notion sets the bar high to define immune correlates of immune intervention therapy: we need to define suitable leukocyte profiles or markers of islet-specific T-cells that could help to assess mechanism of therapeutic action and/or beta-cell preservation.

## Immunopathogenesis of type 1 diabetes

Islet autoreactive CD4 and CD8 T-cells can be detected in blood of T1D patients (4–12). Since their presence is not limited to T1D patients (6, 13, 14), islet autoreactive T-cells cannot be used diagnostically on the individual basis, but changes in their profiles, reactivity and frequency have been shown to correlate with disease and recurrence of T1D after beta-cell replacement therapy. The targets of islet autoreactive T-cells are numerous and include insulin, proinsulin, preproinsulin, GAD65, 38kDa insulin granule protein, ZnT8 and IA-2 (15). Recently, a new universe of modified beta-cell proteins targets was identified as neoantigens in T1D that include post-translational (chemical and enzymatic changes) (16–18) and post-transcriptional modifications (alternative splicing of mRNA and misreads resulting from ribosomal infidelity) (19, 20). Given that these neoantigens are not expected to contribute to thymic education and central immune tolerance, their immunogenicity is high, and their terms of engagement differ from T-cell responses against native self-proteins: T-cells against native autoantigens tend to have lower TCR avidity and docking abnormalities, while their epitope binding affinity to HLA often is low (11, 12, 18, 21–25); T-cells reactive with neoantigens resemble those against viruses, bacteria and alloantigens (17–19, 26, 27). This renders neoantigens strong candidates provoking the immune system and contributing to loss of immune tolerance and epitope spreading that both precede and follow diagnosis of T1D. Neoantigens also point to a role of the target tissue in its own demise, since these proteins changes tend to follow metabolic, inflammatory or infectious stress of pancreatic islets (15).

Efforts to standardize robust immune assays to detect islet-specific autoreactive T-cells have been challenging (28–39). Decades of attempts to standardize T-cells assays by the Immunology of Diabetes Society have led us to appreciate that T-cell autoreactivity does not equal serology in terms of opportunities and expectations to run these assays routinely to robustly measure islet autoimmunity as diagnostics. The frequencies of islet autoreactive CD8 and in particular CD4 T-cells in circulation is very low. Together with the wide range of candidate islet proteins, epitopes and HLA restriction elements, this affects the feasibility to detects islet autoreactive T-cells comprehensively. In case of CD8 T-cell autoreactivity, combinatorial assays were developed to reduce blood volume needs and allow simultaneous detection of different T-cell specificities (7, 28). HLA class II tetramers have rarely been used, and with few HLA-DR polymorphisms and epitopes only (40). No consensus has yet been reached on cryopreservation, ELISPOT and proliferation protocols, but cryopreservation does affect islet autoreactive T-cell responses, notably in case of IL-10 production (41–44). Functional T-cell have been regarded as ‘boutique’ assays that require particular skills and expertise to be executed, thus limiting their use in multicenter clinical trials (3). Detection of islet autoantigen-specific regulatory T-cells (Treg) in clinical blood samples is particularly challenging (45, 46). There are specific Treg phenotypes. Indirectly, the presence of immune regulation of islet autoimmunity can be detected either by IL-10 production by PBMC in response to stimulation with islet autoantigens identified by ELISA (47, 48) or cytokine capture by FACS (10), by cell sorting (10, 49) or limiting dilution analyses (50) showing both effector and regulatory T-cell subpopulations responding to insulin, and by blocking T-cell responses to insulin or GAD65 with anti-HLA-DQ antibodies (51).

This being stated, it does not exclude benefit of T-cell studies to understand disease heterogeneity and progression, and therapeutic or clinical efficacy of immune intervention strategies (8). Given the disappointing outcomes of clinical trials in T1D and the growing awareness of patient and disease heterogeneity, there is an unmet need to define this heterogeneity, mechanism of therapeutic action, responsiveness and clinical efficacy of such trials (52–54). I propose that immune monitoring may offer measures to stratify patients to participate in trials based on immune signatures and genetic barcodes.

## Disease endotypes?

**Figure 1:**
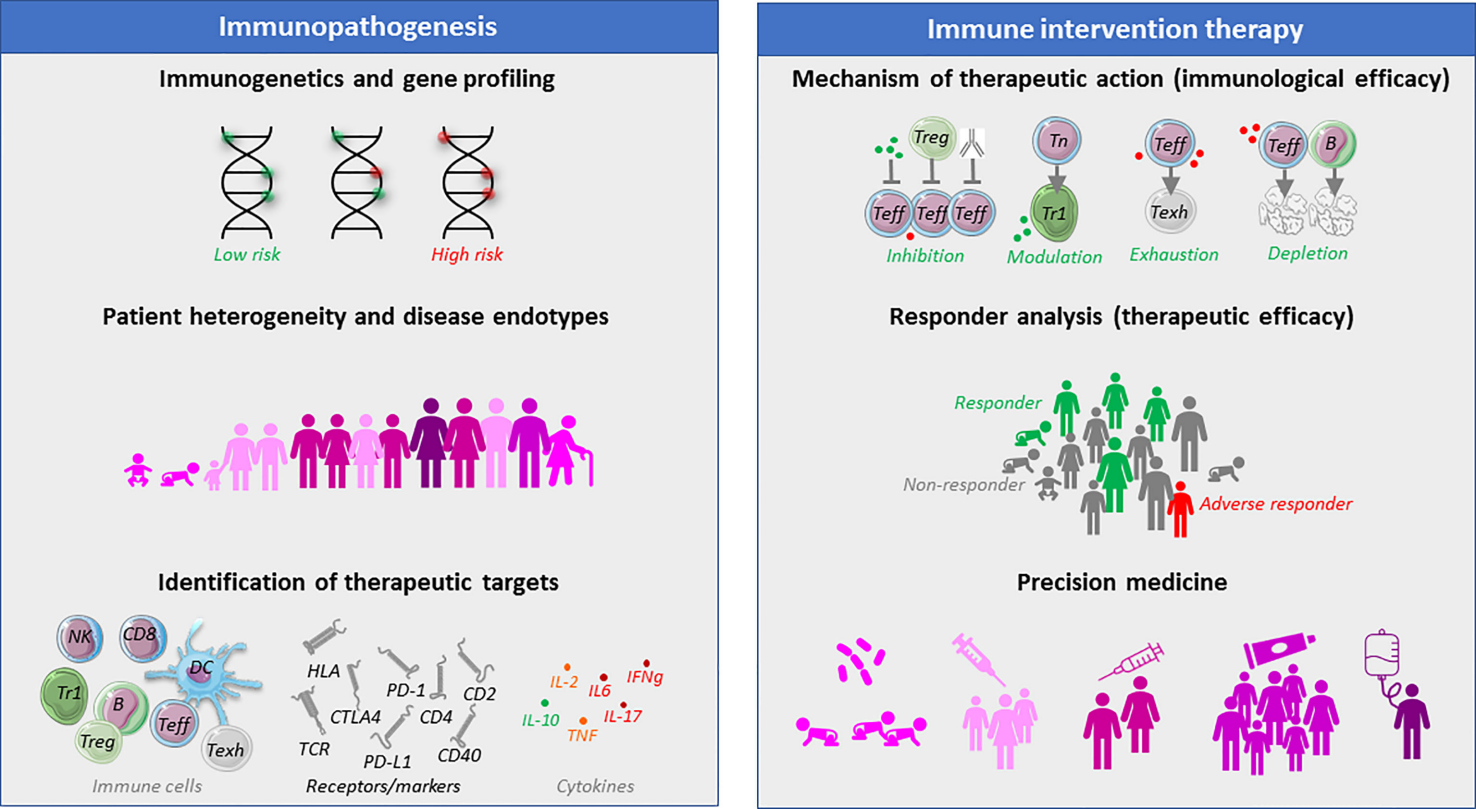
The need and benefit of immune monitoring of T1D patients before and during therapeutic immune intervention. Different aspects of immune monitoring with regard to defining disease heterogeneity, that may lead to the identification of targets of therapeutic intervention. Once such immune intervention is explored in clinical trials, immune monitoring can help define the mechanism of immunological action of an intervention strategy, as well as define baseline characteristics or immunological correlates that may act as endpoints of clinical therapeutic efficacy. It is believed that immune assays may help determine patient subgroups with particular disease entities (endotypes) with particularly favorable chances of clinical benefit (precision medicine), which will avoid subjecting other patients with unlikely clinical benefit to a particular therapeutic intervention strategy.

Genetic diversity between T1D patients is one of the signs that the disease may differ between patients (**Figure 1**) (9, 52, 55). HLA polymorphisms correlate with islet serology and T-cell responses, which is related to the role of HLA in thymic education and antigen presentation. In addition, genetic polymorphisms in for instance IL-2 signaling or vitamin D3 metabolism have implications for efficacy of related immune intervention strategies (56–60). So-called genetic risk scores differ between ancestries and ethnicities, which may reflect different corresponding disease endotypes, as suggested by disease acceleration by abatacept in patients ‘of color’ (ethnicity was not further specified by the authors) versus preservation of beta-cell function in patients of European descent (61–64). Perhaps to most visible correlate of disease variation is age: T1D often presents more aggressively and acutely in infants, whereas disease progression and loss of beta-cell function is more moderate in T1D diagnosed in adults. Insulin autoantibodies are most frequent to first appear in infants below the age of 2, and if they carry HLA-DR4, whereas older children show GAD65 antibodies first before converting to T1D, with HLA-DR3 as genetic correlate (65). The lesion also shows differences with age, with insulitis being moderate in older T1D patients (66). Even in children, two patterns were identified, with children diagnosed before 7 years of age often showed a high rate of inflammation with leukocytes that even included some B cells, whereas children diagnosed beyond 12 years of age show less inflammation and rarely any B-cells (9, 66). This difference in insulitis was mirrored by abnormalities in the remaining beta-cells, with younger cases showing co-localization of proinsulin with insulin, possibly pointing to impaired beta-cell biosynthesis, function and stress. Importantly, T-cell autoreactivity in the pancreatic lesion appears to reflect what is seen in circulation but the reverse is not always true (6, 14, 26, 67–70). Islet autoreactive T-cells isolated from circulation can lyse beta-cells (*i.e.*, diabetogenic) (19, 71), home to pancreatic tissue (72) and cause T1D upon adoptive transfer into humanized mice (73). In terms of T-cell reactivity, distinct profiles of CD4 T-cell autoimmunity could be identified in children with T1D, where some showed immune reactivities to all four tested islet autoantigens (PPI, IA-2, DRiP and GAD65), whereas others responded to none, and half of the children reacted with 2-3 islet autoantigens. Curiously, epitope spreading was most pronounced in children with the longest disease duration, implying activation of new T-cells to new islet autoantigens even after diagnosis. Comparison of T-cell autoreactivity to insulin before and after diagnosis and initiation of insulin replacement therapy revealed a loss of autoreactivity to insulin rather than exacerbation of this response (74). Yet, no autoimmune T-cell correlates could be identified that associated with a temporary clinical remission and reduced insulin need (‘honeymoon’) often seen in the first year after diagnosis of T1D, even though serum cytokine profiles relating to remission were reported (75–77). Autoimmune phenotypes also differed between children and adults, while half of the T1D patients showed signs of IL-17 in response to islet autoantigens (78), underscoring the use of T-cell assays to determine disease heterogeneity and possibly point to endotypes more or less likely to respond to particular intervention therapies. While frequencies of islet autoreactive T-cells often overlap between T1D cases and non-diabetic control subjects, their functionality may differ (48). Intriguingly, T-cell reactivity to islet epitopes was characterized by proinflammatory responses (i.e., IFNγ) in diabetic cases versus anti-inflammatory or regulatory responses (IL-10) in age- and HLA-matched controls. Curiously, patients showing both IFNg and IL-10 in response to islet epitopes manifested disease significantly later than those only producing IFNγ. These observations illustrate the presence of favorable and unfavorable immune signatures that may help identify disease endotypes with faster or slower progression and guide patient selection for distinct therapeutics.

## Identification of therapeutic targets

Immunological monitoring studies have also pointed to potential targets of therapy. For instance, the cytokine production patterns in response to stimulation with islet epitopes has led to the development of several therapeutic strategies aiming to convert a proinflammatory response into an anti-inflammatory one, either with peptides injection in solution, or loaded into tolerogenic dendritic cells or nanoparticles (8, 22, 47, 79–90). The discovery of impaired IL-2 signaling has led to assessment of ultra-low dose IL-2 to selectively stimulate regulatory T-cells (60, 91), while IL-17 production by some patients in response to islet epitopes has made pharma consider to test blockade of this cytokine as intervention strategy (78). The expression of the chemokine receptor CXCR3 by islet infiltrating autoreactive T-cells and the production of the corresponding ligand, chemokine CXCL10 (IP-10), by distressed beta-cells pointed to the opportunity to interfere in leukocyte migration to pancreatic islets (92, 93). A therapeutic monoclonal antibody against B-cells (rituximab) has been tested to preserve beta-cell function shortly after diagnosis (94). Not surprisingly, given my exposé above, this drug only had some effect in the youngest patients. Most importantly, the undisputed role of T-cells in the pathogenesis of T1D has led to the development of several anti-T-cell therapeutic strategies that showed benefit in subsets of patients to either preserve beta-cell function or delay clinical manifestation of the disease (95–97). Additional candidates for targeting by immunotherapy are shown in **Figure 1**.

## Mechanism of therapeutic action (immunological efficacy)

Immunological assays are ideally suited to determine the mechanism of action and potential identify subsets of patients better suited for certain strategies. For instance, in-vitro studies of inhibitors of the co-stimulatory checkpoint CTLA4 that is important in activation and regulation of T-cells responses by antigen-presenting cells proved to have differential effects on naïve T-cells versus autoreactive memory T-cells, impairing activation of the first but sparing reactivation of the latter and implying that this strategy is best suited to prevent priming of naïve T-cells and epitope spreading (63, 98, 99). The effects of anti-thymocyte globulin (ATG) and humanized monoclonal antibodies against CD3 (ChAglyCD3) or CD25 (daclizumab) were tested on prediabetic islet antigen-specific autoreactive T-cells with regard to downmodulation of the target protein, proliferation, cytokine production, complement-dependent cytotoxicity (CDC), antibody-dependent cell cytotoxicity (ADCC), and survival (100). ATG leads to depletion of autoreactive CD4+ T-cells by ADCC, CDC, and apoptosis, whereas anti-CD3 and anti-CD25 inhibited T-cell autoreactivity in a nondepleting fashion. ATG treatment led to a cytokine burst of Th1- and Th2-associated cytokines. Modulation of cytokine release through humanized monoclonal antibodies was moderate and selective: anti-CD25 led to increased release of IL-2 and reduced production of TNF, whereas anti-CD3 decreased release of IFNγ and IL-5 and increased secretion of IL-10. Thus, ATG and the humanized monoclonal antibodies displayed contrasting mechanisms of action.

Ex-vivo analyses of blood samples from participants of immune intervention trials also helped to define the therapeutic mechanism of action. CD19^+^ B lymphocytes were depleted in patients in receiving rituximab (94). In a beautiful side experiment, the authors demonstrated that this B-cell depletion impaired the response to a neo-vaccine, but a subsequent vaccination with the same neoantigen led to immune responses that were indistinguishable from untreated patients, demonstrating that rituximab is a temporary immune suppressant but does not induce immune tolerance (94). Early B cell reconstitution in multiple sclerosis patients treated with rituximab was not associated with a risk of relapse or progression, but instead could reflect regulatory immunological phenomena in subgroup of patients (101). Similarly, T-cell levels temporarily dropped in T1D patients receiving anti-CD3 monoclonal antibodies, abatacept or anti-thymocyte globulin (96, 97, 102, 103). In case of the latter, effector T-cells were hit harder than Tregs, favoring immune regulation. Immunological monitoring of autoimmune and anti-vaccine responses in blood of T1D patients treated with Otelixizumab demonstrated that recall immunity is preserved despite high-dose anti-CD3 treatment, adding to the safety of anti-CD3 treatment as an immune-modulatory agent in the treatment of T1D (104). Reactivation of Epstein-Barr virus and self-limiting mononucleosis-like symptoms were regarded as adverse side-effects but could be seen as evidence of mechanistic efficacy, as this points to the inhibition of active effector T-cells (97). Indeed, CD8 T-cell responses against EBV quickly increased after treatment blunting the clinical symptoms of viral reactivation (102). Blood samples from participants of various islet-antigen specific immune intervention studies showed that injection of islet autoantigen does not exacerbate disease progression but instead induced antigen-specific regulatory T-cells that in some cases corresponded with improved metabolic outcome (8, 79, 81, 83, 105–107). Most mentioned immune modifying intervention therapies caused delays in the fall in C-peptide in subsets of patients but they did not appear to fundamentally alter the immune phenotypes and reactivities durably or change underlying pathophysiology of the disease. This is in line with results from immune monitoring that only showed temporal changes.

## Responder analysis (therapeutic efficacy)

Most value from immunological monitoring came from studies comparing patients clinically responding to certain immune intervention strategies versus those that did not (**Figure 1**). This so-called responder analysis in the rituximab trial revealed that an increased T-cell proliferative responses to islet antigens identified clinical responders to rituximab therapy. In the Teplizumab trial in new-onset T1D a partially exhausted T-cell type (KLRG1^+^TIGIT^+^) was identify, functionally characterize and associated with response to teplizumab therapy (108). Changes in this CD8^+^ T-cell subset of partially exhausted effector cells were associated with clinical response in a trial attempting to delay clinical onset of T1D; these cells showed reduced secretion of IFNγ and TNF (95). While the authors propose that pathways regulating T-cell exhaustion may play a role in successful immune interventions for T1D, biomarkers do not necessarily equal mechanism: the increases in lymphocyte subsets may be a consequence rather than a cause of immunotherapy and reflect relative reductions in other subsets that led to this increase. The teplizumab trial also revealed baseline characteristics that, next to metabolic features, involved a lower frequency of CD4+CCR4+ memory and naïve T-cells, CD4+CCR6+ naïve CD4+ T-cells, naïve CCR4+ CD8+ T-cells, and IFN-γ-producing CD8+ T-cells, versus higher numbers of activated CD8+ terminally differentiated effector and CD8+ effector memory T-cells at baseline in responders versus non-responders. These immune correlates warrant exploring these as predictors of clinical efficacy. Similarly, exhausted-like CD8+ T-cell phenotypes were linked to C-peptide preservation in alefacept-treated (LFA3-Ig) T1D subjects (109), with CD4^+^CD25^+^CD127^hi^ T-cell frequencies at baseline as potential predictor of disease progression and therapeutic efficacy in T1D (110). Autologous hematopoietic stem cell transplantation (aHSCT) is the only therapeutic intervention thus far resulting in complete and sometimes durable remission (insulin independence) in new-onset T1D patients (111, 112). Patients with lower frequencies of autoreactive islet-specific T-cells remained insulin-free longer and presented greater C-peptide levels than those with higher frequencies of these cells (111). In the prolonged-remission-group, baseline islet-specific T-cell autoreactivity persisted after transplantation, but regulatory T-cell counts increased. For the entire follow-up, CD3^+^CD8^+^ T-cell levels did not change, whereas CD3^+^CD4^+^ T-cell numbers remained lower than baseline in both groups, resulting in a CD4/CD8 ratio inversion. Thus, immune monitoring identified a subgroup of patients with superior clinical outcome of aHSCT.

In the context of beta-cell therapies (pancreas, isolated islets or stem cell derived beta-cells), immune monitoring provided seminal proof to determine the in-vivo fate of islet allografts implanted into type 1 diabetic recipients (4, 7, 113–130). In this case, two distinct immune reactions should be monitored: recurrent islet autoimmunity and allograft rejection. Again, baseline immune profiles were highly predictive of clinical outcome: lack of CD4 T-cell autoreactivity to islets resulted in insulin-independence in 86% of the cases, whereas pre-existent T-cell responses to both GAD65 and IA-2 never led to this favored outcome. General T- and B-cell counts also predicted outcome (123, 127). Induction immunotherapy (thymoglobulin, basiliximab or alemtuzumab) led to temporal reductions in T-cell counts and islet autoimmunity that recured in relapsing patients either as isolated phenomenon or followed by allograft rejection, pointing to the pre-existent islet autoimmunity being the principal hurdle in beta-cell replacement therapy in T1D, in spite of continuous immune suppression (tacrolimus, mycomofetyl phenolate and/or sirolimus) that was dictated to prevent induction of alloimmunity to the islet graft. Persistence of recall T-cell immunity against vaccine antigens (e.g., tetanus toxoid) was indicative of sub-maximal or insufficient immune suppression regimes. Immune monitoring also identified recurrent islet autoimmunity as cause of rare and late loss pancreas allograft function in the case of whole pancreas transplantation, underscoring the value of immunological studies (120, 131).

## Future perspectives

The complexity and diversity of the immune system and the range and diversity of immunological features and mechanisms that are believed to be involved in the selective beta-cell destruction leading to T1D put a daunting but indispensable task on the shoulders of immunologists. Despite the many challenges involved, including the shortage of robust and reproducible immune assays and limited access to both blood samples and the lesion, it has proven excessively rewarding to define and apply immune correlates to further define patient and disease heterogeneity, to identify, monitor and validate candidate immune intervention strategies and to define T1D subgroups or endotypes that may most favorable outcome on the one side (precision medicine), and to avoid offering immunotherapies to those unlikely to benefit (‘imprecision medicine’). Immunomonitoring of T1D patients receiving islet allografts defined the immunological fate of the islet grafts *in vivo*, and guided choice of transplant immune suppression, islet encapsulation and gene-editing protocols stem cells and beta-cell progenitors aiming to reduce islet immunogenicity. Given the remaining knowledge gaps in immunopathogenesis and heterogeneity of T1D, it should not be a matter whether, but rather how, to monitor the immune system of T1D patients and at-risk individuals. Bio sampling and storage is critical to determine immunological and possibly even therapeutic efficacy of immune intervention trials. New robust, high through-put and reproducible technologies including multiplex phenotyping and single cell transcriptomics are welcome for the definition of high-resolution immune correlates but given that non-adaptive immune parameters rarely delivered as informative in terms of disease progression, heterogeneity and therapeutic response, focus on the lymphocyte compartment is justifiable. Given that the single endpoint accepted by FDA or EMA as primary outcome remains beta-cell function, whereas the currently explored therapeutic interventions target the immune system rather than beta-cells, there is a growing but largely unmet need to define immune correlates of both mechanistic and clinical efficacy (132). The first steps have been made in this direction and the future looks promising.

## Data availability statement

The original contributions presented in the study are included in the article/Supplementary Material. Further inquiries can be directed to the corresponding author.

## Ethics statement

This report is based on published articles that are referenced. Written informed consent to participate in this study was provided by the participants’ legal guardian/next of kin.

## Author contributions

BR conceived and wrote this perspective. The author confirms being the sole contributor of this work and has approved it for publication.
